# Carotid Revascularization in the Era of Modern Medical Therapy: Is Urgency Still Justified?

**DOI:** 10.1161/SVIN.126.002500

**Published:** 2026-08-12

**Authors:** Calvin D. De Louche, Alun H. Davies

**Affiliations:** Section of Vascular Surgery, Department of Surgery and Cancer, Imperial College London 4615 , United Kingdom.

**Keywords:** Editorials, carotid stenosis, endarterectomy, carotid, ischemic attack, transient, ischemic stroke, secondary prevention, time-to-treatment

After a transient ischemic attack (TIA) or minor stroke, rapid assessment and immediate intensive optimized medical therapy (OMT) should come first. With prompt OMT, many neurologically stable patients with symptomatic carotid stenosis have a low risk of ipsilateral recurrence while awaiting revascularization, suggesting carotid endarterectomy (CEA) can often be planned and delivered selectively, and that OMT alone may be reasonable for some lower-risk groups until trial evidence matures. The timing calculus may differ for transfemoral carotid artery stenting (TF-CAS) and transcarotid artery revascularization (TCAR) because of different antiplatelet requirements and procedural risk profiles, but the central principle remains the same: treat medically now, then revascularize selectively when residual risk justifies it.

## Introduction: The Emergency Is OMT

CEA remains one of the most evidence-based operations in vascular surgery, yet the context that created its landmark stenosis thresholds has changed. CEA was proven to be beneficial during a time when OMT looked very different. Today, intensive antithrombotic regimens, blood pressure control, lipid and diabetes management, and lifestyle modification have reduced the risk of recurrent stroke following TIA/minor stroke, changing the absolute benefit-harm balance of surgery.^1,2^ The practical implication is not simply a higher stenosis threshold, but a different urgency: rapid initiation of OMT, and specialist assessment are essential, whereas the operation itself can often be scheduled rather than rushed. This is particularly true when the patient is neurologically stable and procedural conditions are suboptimal.

This matters at scale. Around 3000 to 3500 CEAs are performed annually in the United Kingdom. In the National Vascular Registry, 30-day death/stroke risk is ≈2.1%, myocardial infarction ≈1.1%, cranial nerve injury ≈1.9%, and bleeding ≈1.8%.^3^ As baseline stroke risk on OMT falls, these upfront hazards loom larger, especially when surgery is forced into suboptimal conditions.

Once intensive OMT is started promptly, the absolute short-term risk of recurrence while awaiting CEA appears far lower than in the trials that created the 14-day paradigm, and hyperacute CEA may increase harm by forcing suboptimal conditions. For many stable patients, CEA should be scheduled or deferred entirely in lower predicted-risk groups, rather than rushed.

This analysis focuses on symptomatic stenosis, drawing on asymptomatic and contemporary trial data to illustrate baseline-risk dilution; the case is for urgent OMT and selective, safe revascularization, not hyperacute CEA (Table).

**Table.**
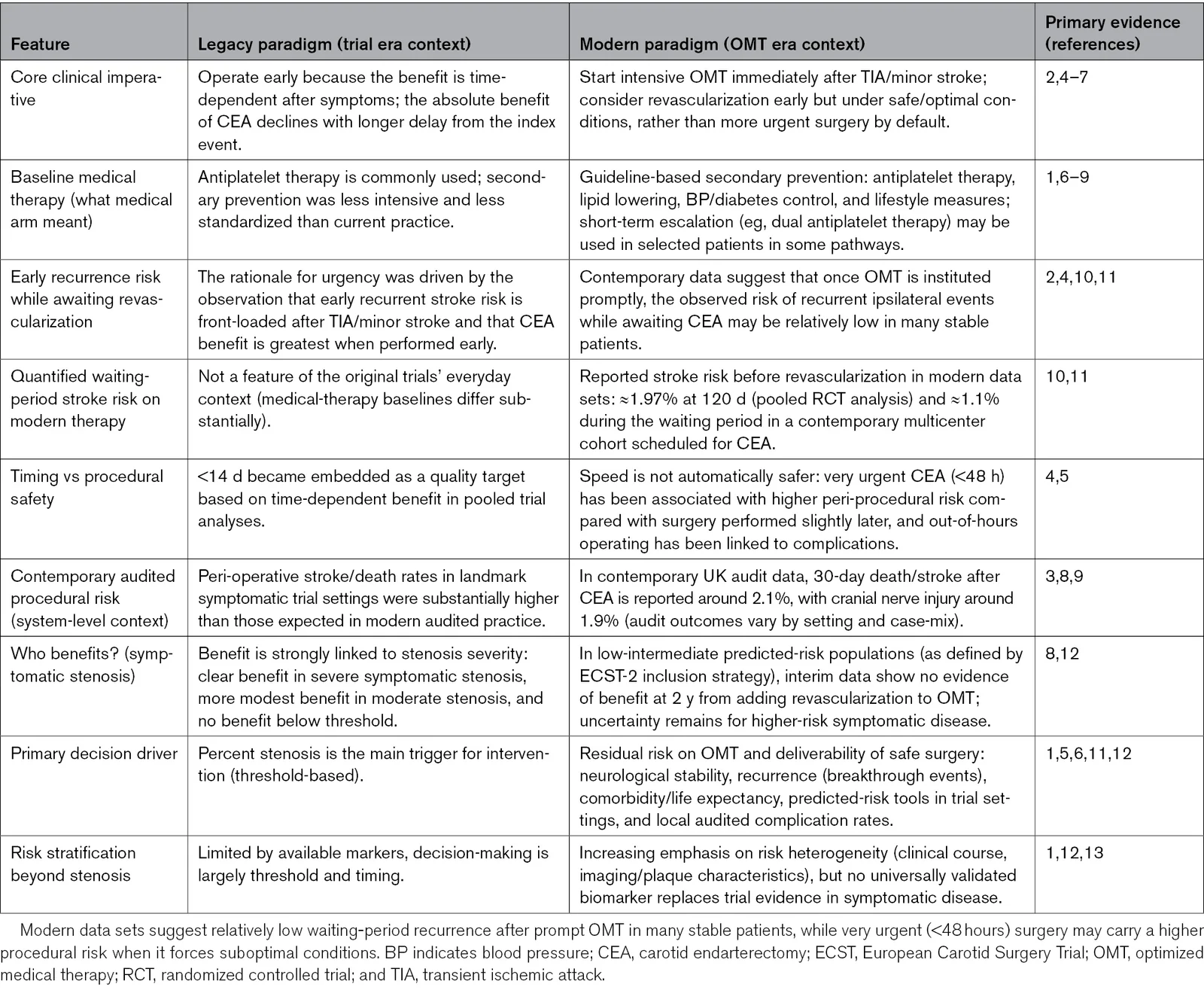
A Summary of the Shift From an Anatomically Threshold-Driven, Time-Pressured Model of Care, Shaped by Landmark Trials, to a Contemporary Approach in Which Immediate OMT and Careful Selection/Timing of Revascularization Are Emphasized

## Why the Classic Urgency Narrative Was Born (and Why It May Not Fit Now)

Historically, the management of carotid artery stenosis has been driven by the perceived relationship between the degree of narrowing and future stroke risk. Landmark randomized trials of CEA versus OMT established the concept of stenosis thresholds, which still shape contemporary guideline recommendations. Classical evidence for such thresholds came from symptomatic-patient trials, including the NASCET (North American Symptomatic Carotid Endarterectomy)^8^ and ECST (European Carotid Surgery Trial),^9^ and in asymptomatic-patient trials, such as the ACST-1 (Asymptomatic Carotid Surgery Trial).^14^ In symptomatic patients with ≥70% stenosis, NASCET reported a marked reduction in ipsilateral stroke risk with CEA, with a durable benefit at 8-year follow-up.^8^ For patients with 50% to 69% stenosis, CEA produced a moderate risk reduction; patients with <50% stenosis did not benefit.^8^ Similarly, ECST demonstrated that benefit was greatest with recently symptomatic severe stenosis and that 30-day peri-operative stroke/death risk (≈7%) needed to be offset against future stroke reduction.^9^ However, these absolute benefits were estimated against medical-therapy baselines from the 1980s to 1990s, before intensive secondary prevention that exists today.

Because ECST and NASCET used different angiographic measurement conventions, modern guideline thresholds are generally interpreted in NASCET-equivalent terms. In ACST, among patients <75 years of age with ≥60% asymptomatic stenosis, successful CEA reduced 10-year stroke risk; however, caution was warranted because the net benefit would depend on future improvements in medical therapy, surgical risk, and life expectancy^14^—an anticipation that better medical therapy could erode the relative advantage of surgery.

The symptomatic trial evidence also established timing as a core determinant of absolute benefit. Legacy pooled analyses of NASCET and ECST data show steep time dependence, underpinning the operate within 14 days culture that is currently adopted.^4^ The recent push for surgery within 2 weeks is grounded in an era with different baseline medical therapy; when intensive OMT is instituted immediately, the assumption that faster is always better becomes less certain, especially if speed compromises procedural conditions. Whether the same urgency applies when early recurrent stroke risk is suppressed by modern OMT is now uncertain. This is exactly what contemporary trials should test, and are beginning to test.

The key question is less about what percent stenosis? and more, what is the residual risk on OMT, and can surgery be delivered (if at all), safely under optimal conditions?

## Modern OMT Changes Early Risk: What Contemporary Data Show While Waiting

Early recurrent stroke risk after TIA/minor stroke drives the rationale for urgent assessment and treatment. In the EXPRESS study (Effect of Urgent Treatment of Transient Ischemic Attack and Minor Stroke on Early Recurrent Stroke), urgent initiation of secondary prevention after TIA/minor stroke was associated with an 80% reduction in 90-day recurrent stroke.^2^

More recent evidence suggests that, once OMT is instituted promptly, the short-term risk while awaiting revascularization may be substantially lower than the historic urgency narrative implies. In a pooled analysis of 4 major modern randomized controlled trials (2000–2008) in symptomatic carotid stenosis, the cumulative stroke risk under medical therapy between randomization and revascularization was 1.97% at 120 days,^10^ much lower than the 5% risk reported in earlier medical-therapy arms in early trials (1980–1990s).^10^

Similarly, a large Danish multicenter registry cohort (2014–2018) provided an especially clinically intuitive message.^11^ Among 1125 symptomatic patients scheduled for CEA, OMT was started at first assessment in 98%, the median delay to surgery was 11 days, and ipsilateral neurological recurrence during the waiting period occurred in 3.6%.^11^ Importantly, stroke-only events were less frequent. Twelve patients had a minor stroke while awaiting CEA, corresponding to ≈1.1% of the scheduled cohort, with no deaths or major strokes while waiting.^11^ These data support the view that immediate OMT followed by early, but not necessarily urgent, CEA was reasonable in high-grade symptomatic stenosis. These temporal changes are summarized graphically in the Figure, which contrasts legacy trial-era medical-therapy risk with contemporary OMT-era waiting-period stroke risk before revascularization.

**Figure.**
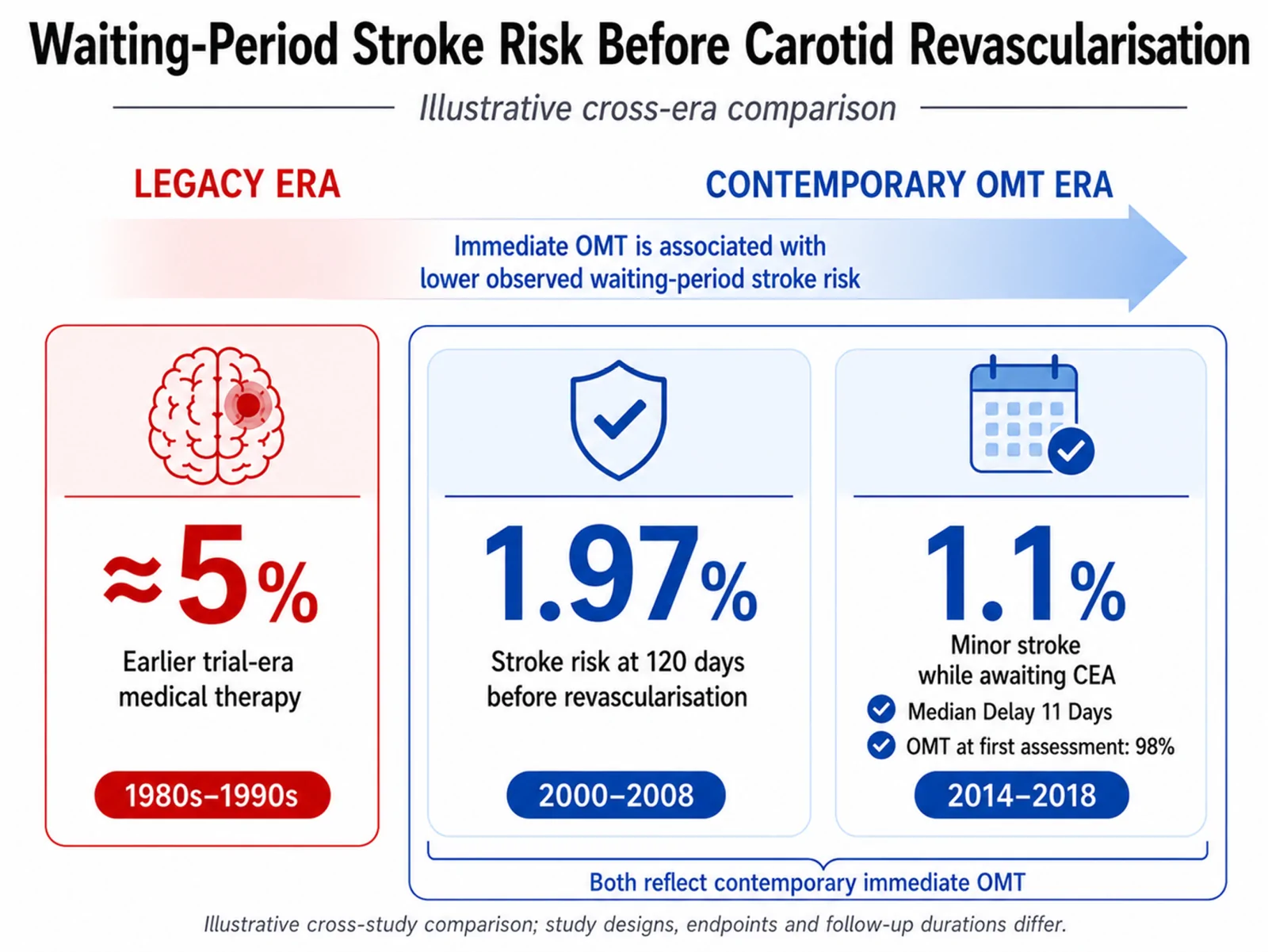
**Illustrative cross-era comparison of reported stroke risk while awaiting carotid revascularization.** Values are not directly comparable because source populations, medical therapy, end points, and follow-up windows differed. The Danish cohort also reported 3.6% ipsilateral neurological recurrence, but stroke-only events were less frequent, with 12 minor strokes among 1125 scheduled patients, ≈1.1%. The figure is intended to illustrate the direction of change in observed waiting-period stroke risk after prompt optimized medical therapy (OMT), not to provide a pooled estimate. CEA indicates carotid endarterectomy.

We suggest that, while a 14-day delivery target may have some advantages to pressurize a healthcare system to deliver care, it can also result in denial of treatment if a patient is deemed to be outside the treatment window, based on day 14 alone. The case for surgery within 48 hours can still be made for a small number of carefully selected patients with recurrent symptoms, high-grade stenosis, or unstable neurology in whom procedural risk remains acceptable despite urgent medical therapy.^4,5^

European guidelines continue to recommend early revascularization after nondisabling symptoms, typically ideally within 2 weeks for symptomatic 50% to 99% stenosis.^6^ This reflects the strong timing gradient seen in the ECST/NASCET era. The 2-week target remains, but in stable patients started promptly on OMT, the necessity for more urgent surgery is less certain than it once appeared.^10^

Clinical teams often convert operating within 14 days into a hard threshold. Importantly, faster is not automatically safer. In the Carotid Alarm Study, CEA within 48 hours was associated with a higher 30-day death/stroke risk than surgery performed between 48 hours and 14 days (8.0% versus 2.9%), and out-of-hours operating was independently associated with complications.^5^ This suggests that when suboptimal operating conditions are forced, the risk of CEA increases significantly.^5^

Intensive antiplatelet therapy is another mechanism by which modern care may buy time. In an expedited-surgery cohort, early dual antiplatelet therapy was associated with fewer recurrent neurological events and reduced spontaneous embolization before CEA, without a clear increase in major peri-operative bleeding in available cohort data, supporting immediate OMT optimization while definitive decisions are made.^7^

## Why Modality Matters

The timing argument should not be assumed to apply identically across revascularization modalities. TF-CAS and TCAR differ from CEA because carotid stenting pathways generally require peri-procedural dual antiplatelet therapy, whereas CEA is often performed on single antiplatelet therapy or carefully selected intensified antiplatelet regimens.^1^ This may, in selected patients, make recent antiplatelet escalation less of a procedural obstacle for stenting than for CEA, although it does not remove the need to balance early recurrent stroke risk against peri-procedural embolic, hemorrhagic, and access-related risks. Current European Stroke Organization guidance states that CAS may be considered in symptomatic patients younger than 70 years with 50% to 99% stenosis, but the recommendation is based on low-quality evidence^6^; European Society for Vascular Surgery guidance recommends dual antiplatelet therapy for patients scheduled for carotid stenting.^1^ TCAR has reported favorable early outcomes in single-arm registry data: ROADSTER-2 (Post-Approval Study of Transcarotid Artery Revascularization in Patients With Significant Carotid Artery Disease) reported 30-day stroke/death of 2.3% and stroke/death/myocardial infarction of 3.2% in the intent-to-treat population, but this was not a randomized comparison against contemporary OMT or CEA.^15^ Therefore, our conclusion should be read as modality-conscious rather than CEA-exclusive.

Importantly, CEA remains the principal evidence base for the timing discussion, while TF-CAS and TCAR are considered separately because their antiplatelet requirements, anatomic constraints, and peri-procedural risk profiles differ from CEA. Immediate OMT is the emergency; subsequent CEA, TF-CAS, or TCAR should be selected according to residual stroke risk, anatomy, age, antiplatelet tolerance, operator outcomes, and the ability to deliver the procedure safely.

## Modern Randomized Data Challenge: Default Revascularization in Lower-Risk Groups

ECST-2, the successor of ECST, is an ongoing multicenter randomized controlled trial recruiting patients with ≥50% carotid stenosis (either symptomatic or asymptomatic) with low to intermediate predicted stroke risk based on the carotid artery risk score, assigning them to either OMT alone or OMT plus revascularization (CEA or stenting).^12^ Of note, ECST-2 does not resolve the remaining uncertainty in higher-risk symptomatic disease.^12^

In the published 2-year interim analysis, there was no evidence of benefit from adding revascularization to OMT, and the authors explicitly state that the results support treating patients with asymptomatic and low/intermediate-risk symptomatic stenosis with OMT alone until longer-term results are available.^12^ Hence, for patients whose predicted stroke risk is already low on OMT (the ECST-2 population), the incremental benefit of adding CEA or CAS appears small at 2 years, raising the threshold at which surgery is justified. Whether this lack of observed benefit persists at longer follow-up (5 years and beyond) remains uncertain.^12^

If a sizeable symptomatic subgroup derives little early incremental benefit from revascularization over OMT, then in stable patients the default pathway should be immediate intensive OMT with planned surgery only when residual risk remains convincingly high.

This reflects a broader phenomenon in carotid disease, namely baseline-risk dilution. In contemporary cohorts of medically treated asymptomatic carotid stenosis, ipsilateral stroke rates appear low for many patients and have fallen over time, but risk is heterogeneous and increases with stenosis severity and other determinants.^16,17^ Randomized evidence in asymptomatic high-grade stenosis illustrates how improved medical therapy compresses absolute event rates and may produce modality-specific conclusions. In CREST-2, patients with high-grade asymptomatic carotid stenosis were enrolled into 2 parallel trials comparing intensive medical management alone with either carotid-artery stenting plus intensive medical management or CEA plus intensive medical management.^18^ At 4 years, stenting plus intensive medical management reduced the composite outcome compared with intensive medical management alone, whereas CEA plus intensive medical management did not show a statistically significant benefit over intensive medical management alone.^18^ These results should not be directly transferred to recently symptomatic stenosis, but they do reinforce the need to analyze CEA, TF-CAS, and TCAR separately rather than treating revascularization as a single intervention. They also strengthen, rather than weaken, the central premise of our argument that modern OMT has lowered baseline event rates enough that the incremental benefit of any procedure must be demonstrated in contemporary, modality-specific populations.

The critical remaining question is whether patients with high-grade (≥70% NASCET-equivalent) symptomatic stenosis, optimized on contemporary OMT, still derive enough incremental benefit from CEA to justify expedited intervention. This is an area where further randomized evidence is needed.

## Pragmatic Risk-Based Pathway: Urgent OMT for All; Selective Timing of Revascularization

Regardless of whether CEA is ultimately performed, the first-line, time-critical intervention is intensive OMT started immediately at the first assessment. This includes antiplatelet treatment, statins, and rigorous control of blood pressure, lipids, and lifestyle risk factors, as emphasized by contemporary guidelines and reviews.^1,6^

A central practical problem is that there is no validated biomarker that reliably identifies who will or will not benefit from CEA in the setting of contemporary OMT. In symptomatic disease, decision-making therefore still relies on pragmatic surrogates: neurological stability, recurrence or breakthrough events on OMT, comorbidity/life expectancy, and whether surgery can be delivered with low audited complication rates. In asymptomatic stenosis, embolic signals, plaque morphology, cross-sectional imaging, and silent infarction have been explored as risk markers, but none fully substitutes for randomized evidence in modern practice.

One pragmatic way to operationalize this in stable symptomatic stenosis might be:

- Day 0 to 1: diagnose, start intensive OMT immediately, optimize blood pressure, lipid status, and antithrombotic therapy, specialist stroke/TIA unit care.^11^
- Day 1 to 7: risk-stratify (recurrent symptoms on OMT, plaque features, physiology, comorbidity, local audited complication rates).
- Thereafter: plan modality-specific revascularization (CEA, TF-CAS, or TCAR) electively, if at all, rather than rushing intervention; urgent intervention remains appropriate when instability or recurrent events indicate high residual risk and when procedural risk is acceptable.^11^

By contrast, for low- or intermediate-risk symptomatic patients and most patients with asymptomatic disease, CEA should be reserved for carefully selected individuals with convincing markers of higher risk of future stroke as opposed to being offered simply because of a percentage cutoff.^17^

## What We Still Don’t Know: Practice Change Requires Contemporary Randomized Evidence

Ongoing trials and further cohort work, including detailed analyses of temporal trends and plaque-related risk, will be important for refining risk prediction and identifying any subgroups for whom intervention would remain advantageous.^13,16^ When considering health economics, the combination of falling medical-arm risks and nontrivial perioperative event rates raises legitimate questions about the cost-effectiveness and appropriateness of high-volume CEA programs for moderate symptomatic stenosis,^19^ but also for unselected asymptomatic patients.^16,20^ Recent modeling suggests that CEA may no longer be clearly clinically effective or cost-effective in some moderate symptomatic cohorts, and explicitly calls for randomized evidence in this space.^19^

Future trials will need to focus on precisely those subgroups whose residual risk on contemporary OMT is high enough to justify intervention. Until such evidence is available, this article should not be read as a call for wholesale abandonment of early revascularization in symptomatic severe stenosis. Rather, it argues for a safer and more evidence-sensitive sequence: immediate OMT for all, urgent intervention for unstable or recurrently symptomatic patients, and planned selective revascularization for neurologically stable patients when residual risk remains high, and the procedure can be delivered under optimal conditions. A definitive change in practice for symptomatic ≥70% stenosis requires adequately powered randomized controlled trials comparing contemporary OMT alone with OMT plus modality-specific revascularization, including CEA, TF-CAS, and TCAR.

## Conclusions: Deemphasize the Rush

In contemporary practice, the priority after TIA/minor stroke is rapid diagnosis and immediate intensive secondary prevention. Contemporary data sets suggest that, once OMT is instituted promptly, many stable patients experience a low short-term recurrence risk while awaiting revascularization, and hyperacute surgery may increase complications when it forces suboptimal conditions. ECST-2 interim results further suggest that, in patients with low to intermediate predicted risk, adding revascularization to OMT may offer little early advantage. CEA remains important for selected high-risk patients, particularly those with recurrent events despite OMT, but TF-CAS and TCAR require separate consideration because their antiplatelet requirements, anatomic constraints, and peri-procedural risk profiles differ from CEA. CREST-2 reinforces this modality-specific point in asymptomatic disease, but it does not settle the question in recently symptomatic severe stenosis.

The pressing question is no longer “How fast can we operate?” but rather: “After immediate OMT, who still has enough residual risk to justify revascularization, by which modality, and when can it be delivered under the safest conditions?”

## ARTICLE INFORMATION

### Author Contributions

C.D. De Louche (Academic Foundation Doctor and Honorary Clinical Research Assistant, Department of Surgery & Cancer, Imperial College London) undertook the literature search, extracted key data from trials, guidelines, and contemporary observational studies, and wrote the first draft. A.H. Davies (Professor of Vascular Surgery, Imperial College London; Consultant Vascular Surgeon at Imperial College Healthcare NHS Trust) conceived the article’s central argument, provided senior clinical interpretation, and critically revised the article for intellectual content and international relevance. Sources used included landmark randomized trials of carotid endarterectomy, contemporary European guidelines, and recent randomized and observational evidence evaluating outcomes with optimized medical therapy and revascularization. The guarantor is A.H. Davies.

### Disclosures

None.
